# The Role of Extra Corporeal Membrane Oxygenation in Amniotic Fluid Embolism: A Case Report and Literature Review

**DOI:** 10.7759/cureus.13566

**Published:** 2021-02-26

**Authors:** Shalini Durgam, Munish Sharma, Rahul Dadhwal, Abhay Vakil, Salim Surani

**Affiliations:** 1 Internal Medicine, Corpus Christi Medical Center, Corpus Christi, USA; 2 Pulmonary Medicine, Corpus Christi Medical Center, Corpus Christi, USA; 3 Internal Medicine, University of North Texas, Denton, USA; 4 Internal Medicine, University of North Texas, Dallas, USA

**Keywords:** amniotic fluid embolism, labor and delivery, extra corporeal membrane oxygenation, hypoxic respiratory failure, right ventricular failure, acute respiratory distress syndrome

## Abstract

Amniotic fluid embolism (AFE) is a rare and life-threatening complication related to pregnancy. Early diagnosis and prompt intervention are important tools for the survival of the patient. Despite early intervention, mortality rate remains high. We present a case of a 19-year-old female who was admitted for labor induction and delivery. Her delivery course was complicated by meconium-stained amniotic fluid. The patient went into acute hypoxic respiratory failure (AHRF) and hemodynamic compromise within half an hour following delivery secondary to AFE. We hereby discuss the role of timely initiation of extra corporeal membrane oxygenation (ECMO) in a case of AFE which could have otherwise turned out to be fatal.

## Introduction

Amniotic fluid embolism (AFE) is a rare clinical entity that occurs when amniotic fluid enters the maternal circulation. It is estimated to have an incidence ranging from 1.9 to 6.1 cases /100,000 deliveries as per a combined data obtained from Australia, Canada, United States of America, the United Kingdom, and the Netherlands [1]. This might even reflect an overestimation as a large fraction of patients might just be assumed to have AFE based on nonspecific findings [2]. AFE is reported to cause 10% of total maternal deaths even in the developed countries [3]. There are older reports suggesting up to 90% maternal mortality while the newer evidence suggest around 50% mortality [4-5]. It is estimated, to have a relatively better outcome, there should not be more than four minutes between cardiopulmonary collapse and delivery of the fetus [6]. Thus, management of AFE is extremely time sensitive and institution of appropriate life-saving measures should be initiated promptly and determined on a case-by-case basis. Here we describe the use of veno-arterial (VA) extra corporeal membrane oxygenation (ECMO) for acute hypoxic respiratory failure (AHRF) and acute right ventricular (RV) failure due to AFE.

## Case presentation

**Figure 1:**
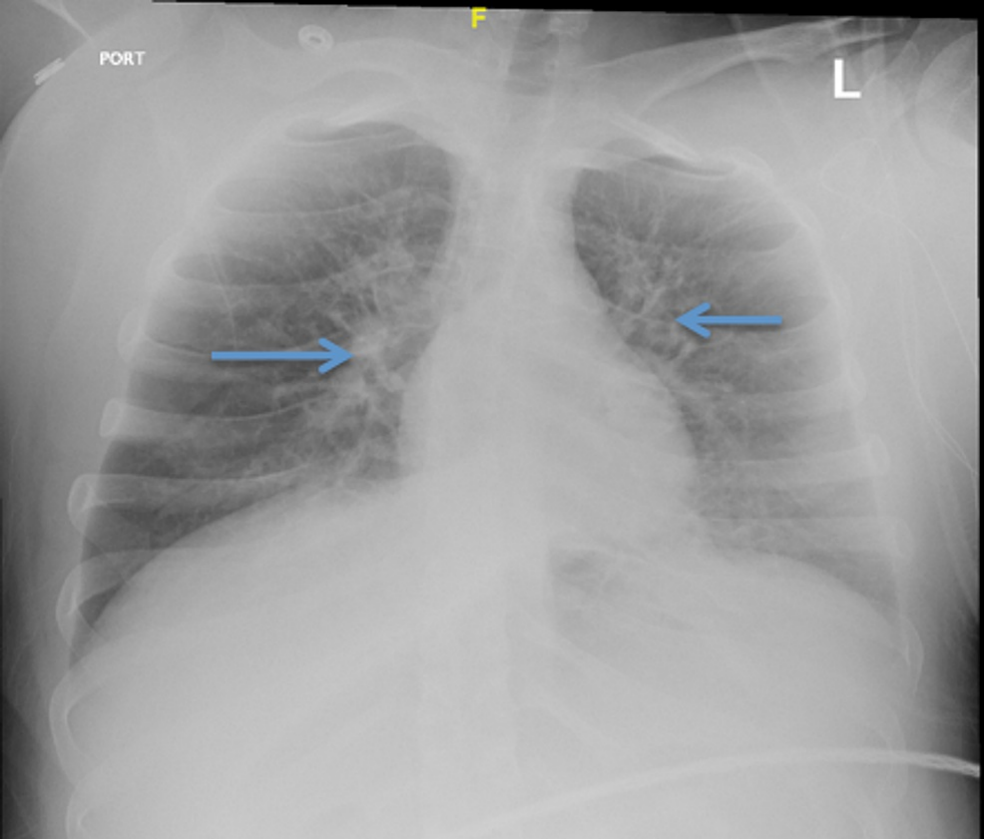
Pre-intubation chest X-ray showing bilateral infiltrates mainly in the perihilar regions (blue arrows).

A 19-year-old female (primigravida and nulliparous) with no known past medical history was admitted to the hospital at 40 weeks 5 days of gestational age for induction of labor and delivery. The patient had regular prenatal visits and all laboratory workup and fetal ultra-sonogram were done according to the gestational age and were unremarkable. Labor was induced with oxytocin and cervical misoprostol. The patient had a temperature of 102.2^o^F one hour before the delivery and was given one dose of acetaminophen 650 mg which helped in controlling the fever. She had stable vital signs otherwise. The patient had a normal vaginal delivery. Amniotic fluid was meconium stained. The infant had an APGAR score of 1 at one minute and a score of 6 after five minutes. After about half an hour of delivery, the patient became dyspneic with a respiratory rate of 30 breaths/minute, blood pressure of 82/52 mmHg, heart rate of 150 beats/minute, and oxygen saturation of 37% on room air. A rapid response was initiated (also known as urgent response team) and the patient was transferred to the ICU. A chest X-ray showed bilateral infiltrates (Figure 1).

She was tried and failed the bilevel noninvasive ventilation for around 10 minutes and was immediately intubated and placed on the mechanical ventilation. She was sedated with fentanyl, propofol, and dexmedetomidine. Arterial blood gas (ABG) findings after intubation, pH was normalized to 7.39, pCO2 43.4, pO2 83.3, HCO3 25.9, vent rate 30/min, fiO2 100%, tidal volume 350 mL/breath, and PEEP 5 cm H2O. In view of persistent hypotension even after isotonic fluid bolus challenge, the patient had to be maintained on multiple vasopressor drips that included norepinephrine, phenylephrine, vasopressin, and epinephrine. Broad-spectrum empiric antibiotics coverage such as linezolid, cefepime, and metronidazole were also started. Complete blood count showed white blood cell (WBC) 24.5/uL (4.8-10.8), red blood cell (RBC) 2.21/uL (4.2-5.4), hemoglobin (Hgb) 7 g/dL (12-16), hematocrit 20.9% (37-47), and platelet 76/uL (150-450). Basic metabolic panel (BMP) showed sodium 139 mmol/L (133-145), potassium 3.1 mmol/L (3.6-5.2), chloride 104 mmol/L (100-108), CO2 mmol/L14 (22-32), blood urea nitrogen (BUN) 12 mg/dL (16-20), creatinine 1.86 mg/dL (0.6-1), lactic acid 15.6 mmol/L (0.5-2.2), calcium 7.5 (8.7-10.5); liver function tests revealed total bilirubin 2.9 mg/dL (0-1.0), aspartate aminotransferase (AST) 69 units/L (15-37), alanine aminotransferase (ALT) 9 units/L (30-65), and alkaline phosphate 834 units/L (50-130). Coagulation studies showed elevated prothrombin time (PT) 19.5 seconds (reference range 9.6-12.3), activated partial thromboplastin time (aPTT) 36.2 seconds (22.5-35.3), fibrinogen 144 mg/dL (200-400), INR 1.71, D-dimer > 7650 ng/mL FEU (<500). Initial disseminated intravascular coagulation (DIC) score was 7. The patient received three units of packed red blood cells (PRBC), two units fresh frozen plasma (FFP), two units cryoprecipitate, and two units platelets.

**Figure 2:**
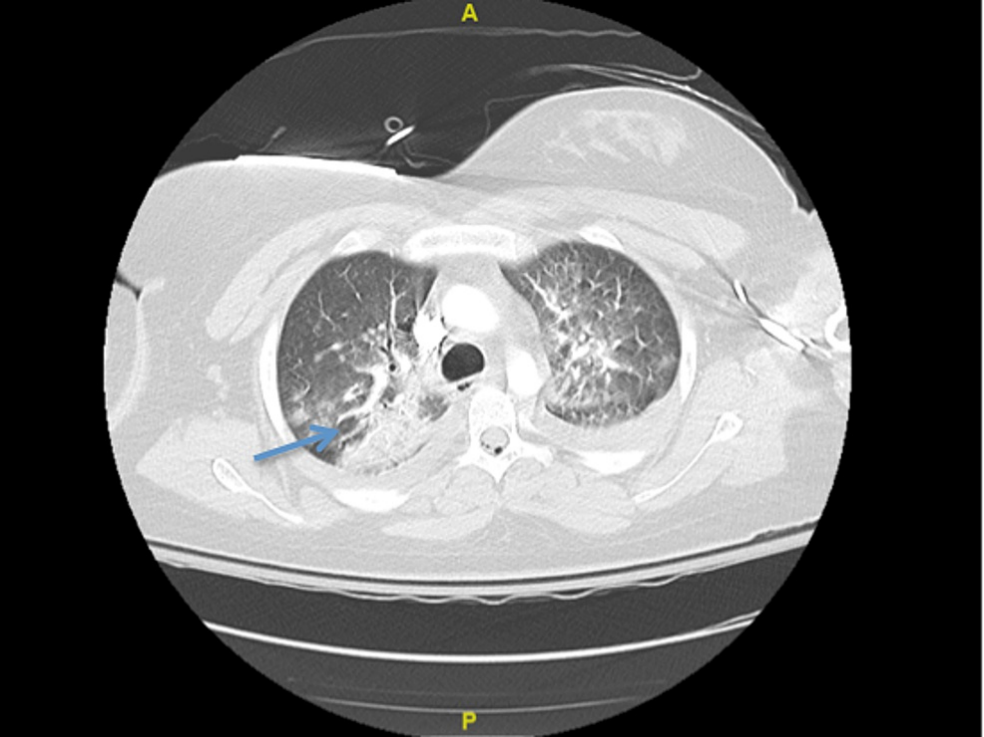
CT angiogram of the chest concerning for AFE (blue arrow). AFE, amniotic fluid embolism

**Figure 3:**
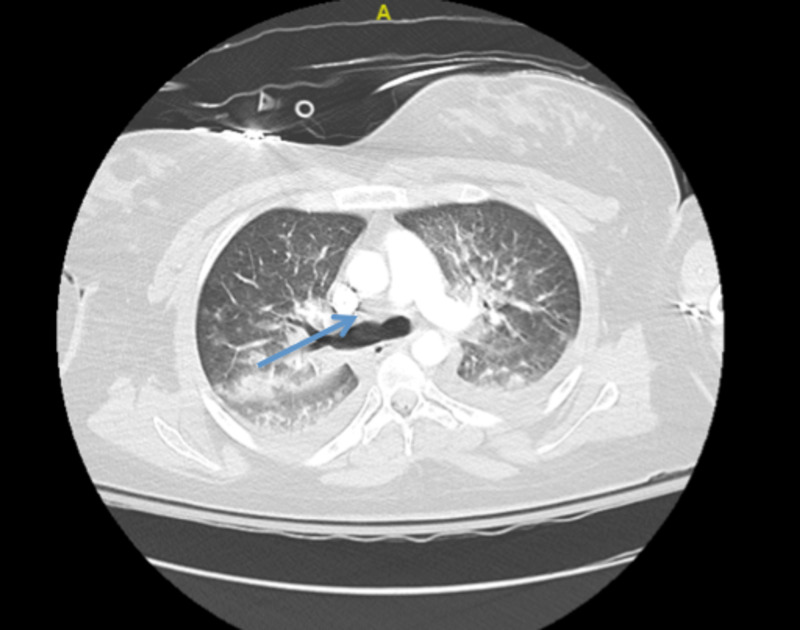
CT angiogram of the chest concerning for AFE (blue arrow). AFE, amniotic fluid embolism

Initial troponin was 2.11 ng/mL which later increased to 5.96 ng/mL. CT angiogram chest showed small embolus in the right upper lobe and findings were concerning for AFE (Figures 2-3).

 

The patient had an urgent bedside transthoracic echocardiogram (TTE) that showed ejection fraction (EF) > 70%, no regional wall motion abnormalities, normal diastolic function parameters, right ventricle dilation, reduced systolic function, systolic pressure severely increased in pulmonary arteries, estimated to be 65 mmHg. The TAPSE was not calculated. Given the patient’s critical condition, it was planned to send the patient to a higher center for ECMO support. The patient was placed on vena-arterial (VA) ECMO via a bi-femoral approach. The patient sustained a brief episode of pulseless electrical activity (PEA) cardiac arrest requiring one dose of epinephrine and 2 minutes of high-quality chest compression with the return of spontaneous circulation (ROSC). Postcannulation of VA ECMO, the patient required additional six units PRBC, one unit of platelet along with supplementation of calcium gluconate and sodium bicarbonate. The patient was on low-dose epinephrine that was eventually weaned off within day 2 of delivery. The patient was extubated on the second day. Hematology was consulted for coagulopathy with disseminated intravascular coagulation (DIC) that was deemed secondary to shock liver. As low antithrombin III levels may have decreased the effectiveness of heparin, she was given low dose bivalirudin bolus for ECMO continuation. Later the patient was transitioned to warfarin with enoxaparin bridging on discharge.

The patient was also noted to have anuric acute renal failure and elevated lactate with a high anion gap metabolic acidosis and hypernatremia. Nephrology initiated continuous renal replacement therapy (CRRT) which was discontinued before discharge with normalization of renal functions. The patient was weaned off ECMO on day 4. The patient's condition progressively stabilized and was discharged from the hospital on day 8.

## Discussion

Amniotic fluid embolism is one rare but life-threatening complication related to pregnancy, wherein amniotic fluid will enter into maternal pulmonary circulation leading to cardiovascular collapse. It can occur during amniocentesis, medical induction of labor, following vaginal, cesarean section, 48 hours after postpartum [7]. AFE was reported as early as in 1926 by two pathologists Steiner and Lauschbaug. They reported 32 cases of women dying of obstetric shock during labor [8]. Pathophysiology is not clearly defined. It was hypothesized in some studies that it was due to maternal/fetal interface breach leading to immune activation and release of pro inflammatory mediators and vasoactive agents representing similar to systemic inflammatory response syndrome and termed as anaphylactoid reaction of pregnancy [2]. Risk factors associated with the development of AFE are amniocentesis, placenta previa, medical induction of labor, advanced maternal age, increased fetal gestational age, male fetus, cesarean section, multiparity, multiple pregnancies, and trauma [3-5]. AFE can present with signs and symptoms of hypoxia, hypotension, dyspnea, fever, chills, fetal distress, DIC as presented in our case scenario. The potential for mortality is increased with meconium stained amniotic fluid [4]. AFE is a diagnosis of exclusion. Early diagnosis and management improve the survival. Early diagnostic tools that help in the confirmation of AFE are blood counts, chest X-ray, D-dimer, CT chest, ABG, coagulation studies, and echocardiogram. Diagnostic criteria, proposed by Society for Maternal-Fetal Medicine (SMFM) and Amniotic Fluid Embolism Foundation for diagnosis of AFE are shown in Table 1. Presence of all four of the following diagnostic criteria should be there for diagnosis of AFE [9].

**Table 1:** Diagnostic criteria for AFE. AFE, amniotic fluid embolism; DIC, disseminated intravascular coagulation

1.Cardio-respiratory arrest with evidence of hemodynamic compromise
2.Clinical onset during labor or within half hour following placental delivery
3. DIC and score of ≥3 is compatible with overt DIC
4. Absence of fever (≥38°C) during labor

Management of AFE is supportive. During oxygen supplementation to prevent hypoxia, oxygen can be delivered through nasal cannula, nonrebreather mask, BiPAP, and endotracheal intubation. Hemodynamic stability can be achieved with fluid resuscitation; patients who have refractory hypotension can be started on pressor support. Hemorrhage can be managed with blood transfusion and coagulation abnormalities can be managed with cryoprecipitate, FFPs and in some cases with massive transfusion protocol [10].

In patients with refractory cardiovascular collapse, ECMO is the important modality of choice [11]. There are only few articles which discuss about the application of ECMO in AFE patients. ECMO is of two types namely veno arterial (VA) and veno-venous (VV). VA ECMO provides cardiovascular and gaseous exchange support and VV ECMO provides only gaseous support. VA ECMO was used in our case. Indications for VA ECMO include low cardiac output (cardiac index less 2 L/min/m2) and hypotension (systolic blood pressure <90 mmHg) despite adequate volume resuscitation, high dose inotropic agents, and intra-aortic balloon pump [12]. Early diagnosis of patients with AFE with bedside echo and early intervention of ECMO in refractory cases helps in improving oxygenation and decreasing cardiac workload [13]. Anticoagulation should be initiated with ECMO to prevent complications like systemic thromboembolism and circuit thrombosis. In our case, we did not initiate anticoagulation initially due to DIC but as coagulopathy improved, anticoagulation was initiated.

The main goal of ECMO is to achieve hemodynamic stability. As in our case, the patient was in severe hemodynamic compromise, DIC, requiring massive transfusion protocol and was refractory to vasopressor support going into cardiac arrest where early intervention with ECMO led to a successful outcome. Not only prompt intervention but also early weaning of ECMO is important, as complications like hemolysis, renal function impairment, intracranial hemorrhage, sepsis, and limb ischemia are directly related to the duration of the extracorporeal life support [14-15].

## Conclusions

Amniotic fluid embolism is a rarely encountered clinical entity but it is known to have devastating outcomes. Early diagnosis and initiation of resuscitative measures can prove pivotal in recovery of the patient. Catastrophic coagulation disorders such as DIC, severe hypoxic respiratory failure, and sudden onset cardiac failure play a central role in heralding life-threatening consequences. Though the management in AFE may vary on a case-by-case basis, early initiation of VA ECMO proved extremely important for complete recovery of our patient. There is not much literature regarding the role of VA ECMO in AFE and reports such as this will be important in adding to the current literature.
